# Risk of malignant disease in 1-year sepsis survivors, a registry-based nationwide follow-up study

**DOI:** 10.1186/s13054-023-04654-9

**Published:** 2023-09-29

**Authors:** Johanna Hästbacka, Anna But, Gunnar Strandberg, Miklós Lipcsey

**Affiliations:** 1 Department of Intensive Care, Tampere University, Faculty of Medicine and Health Technology, and Tampere University Hospital, Tampere, Finland; 2 Biostatistics Consulting, Department of Public Health, Helsinki University Hospital, University of Helsinki, Helsinki, Finland; 3 Section of Anesthesiology and Intensive Care, Department of Surgical Sciences, Uppsala University Hospital, Uppsala, Sweden; 4 Hedenstierna Laboratory, Department of Surgical Science, Uppsala University, Uppsala, Sweden

**Keywords:** Sepsis, Cancer, Long-term outcome

## Abstract

**Background:**

Cancer and sepsis share risk factors, and sepsis patients may have impaired immune response and increased morbidity long after intensive care. This study aimed to assess whether sepsis survivors are at increased risk for cancer. Our objective was to assess the incidence of new cancer in 1-year sepsis survivors and test the hypothesis that it is higher than that of the general population.

**Methods:**

We obtained data on ICU admissions of adult patients from Swedish Intensive care registry (SICR) from 2005 to 2017. We included patients with an explicit ICD-10 code for sepsis for the primary ICU admission. We obtained data on cancer diagnoses (2001–2018), death (2005–2018) and emigration (2005–2018) from Cancer and Cause of death and National Patient Registry databases of the National Board of Health and Welfare; age and sex-specific cancer incidence rates in Sweden from NORDCAN registry from 2006 to 2018. One-year survivors formed the final cohort, that was followed for new cancer diagnoses until death, emigration, or end of 2018, whichever came first. The main outcome measure was standardized incidence rate ratio (SIR) to compare the incidence of cancer in 1-year sepsis survivors to that in the general population (NORDCAN). We also performed several sensitivity analyses.

**Results:**

In a cohort of 18,550 1-year survivors, 75,427 person years accumulated during a median follow-up (FU) of 3.36 years (IQR 1.72–5.86), 6366 (34.3%) patients died, and 1625 (8.8%) patients were diagnosed with a new cancer after a median FU of 2.51 (IQR 1.09–4.48) years. The incidence ratio of any new cancer over the whole FU was 1.31 (95% CI 1.23–1.40) for men and 1.74 (95% CI 1.61–1.88) for women. The difference in incidence rates persisted in several sensitivity analyses. The SIRs were highest in cancers of gastrointestinal tract, genital organs, and skin.

**Conclusion and relevance:**

Compared to general population, incidence of cancer is increased in 1-year sepsis survivors. Variation in the findings depending on follow-up time suggests that factors other than sepsis alone are involved. Surveillance for malignant disease may be warranted in sepsis survivors.

**Graphical abstract:**

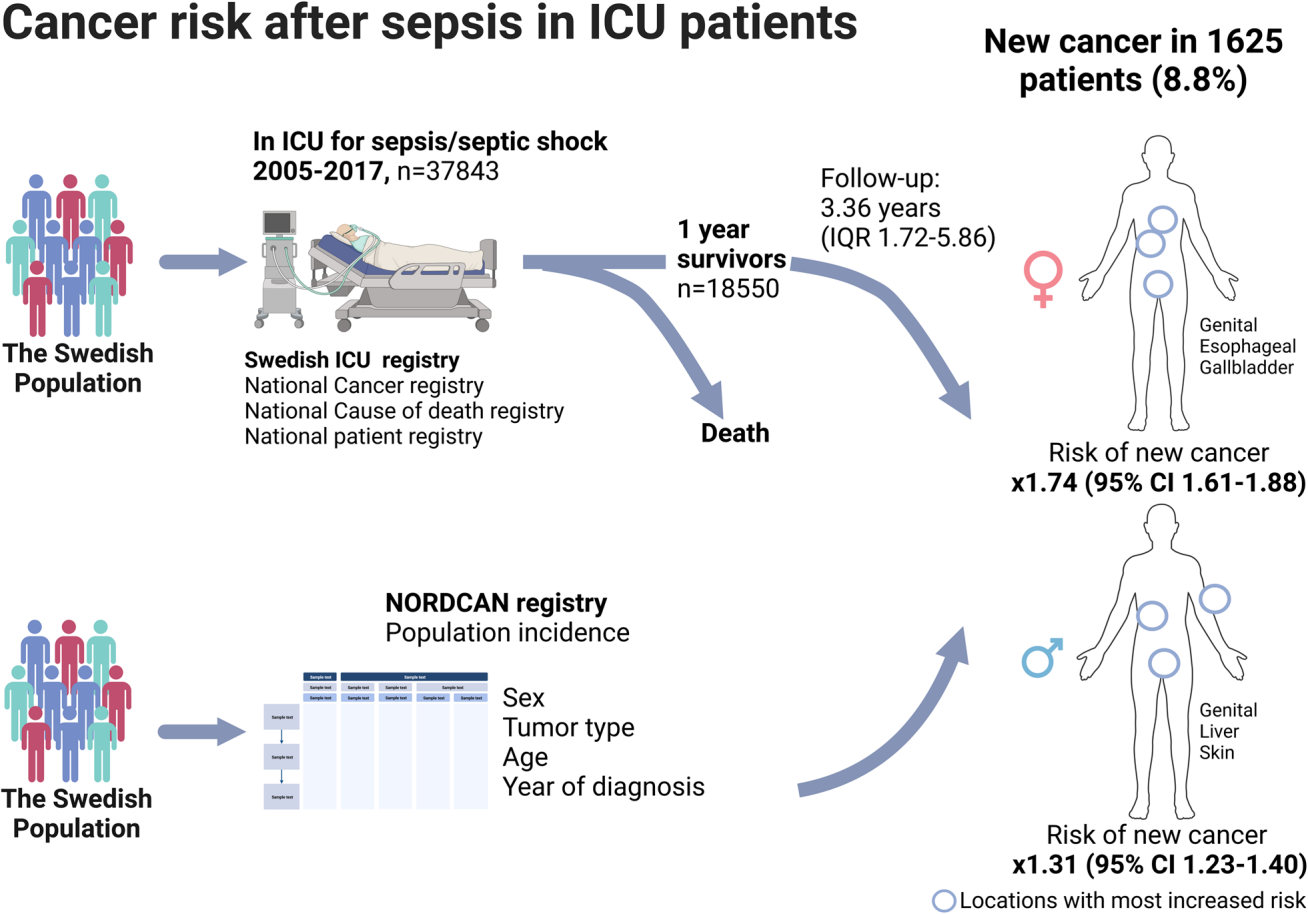

**Supplementary Information:**

The online version contains supplementary material available at 10.1186/s13054-023-04654-9.

## Background

Sepsis patients are at increased risk of death long after their intensive care unit (ICU) treatment compared to non-septic critically ill patients and the general population [1, 2]. This risk also concerns younger sepsis patients with no comorbidities [1]. Data on long-term outcomes of sepsis patients are still scarce. Malignant disease is common comorbidity in patients admitted to ICU for sepsis and has been reported as cause of death in nearly 50% of sepsis patients dying after discharged alive from hospital [3, 4]. While chronic health status is a major determinant of long-term outcomes [5], severe acute disease such as sepsis may reduce the overall physiological reserves of ICU survivors and lead to poor long-term recovery [6–10].

Sepsis patients surviving the initial inflammatory response may enter a chronic state of impaired immune response [11], which may impact long-term morbidity. Apoptosis of lymphatic tissue has been demonstrated in autopsy of sepsis non-survivors [12, 13], and sepsis survivors with impaired immune function are susceptible to infections, the most common reasons for readmission after sepsis [14–16]. Theoretically, this could also contribute to the risk for malignant disease, among other factors [17]. Impaired immune surveillance after sepsis promoted tumor growth in an experimental animal model of polymicrobial sepsis [18]. Moreover, persistent inflammation may increase the risk for malignant growth [19]. Compromised immune function associates with malignant development in human immune deficiency (HIV) patients and solid organ transplantation recipients, who have an increased risk for certain cancer types [20–23]. The patterns of increased risk for cancer in those two patient groups are similar, suggesting that immune dysfunction as an etiological risk factor may be relevant [24].

Recently, an association between former sepsis and certain malignancies was found in a registry-based study from the United States [25]. By matching elderly patients with first cancer diagnosis with controls without cancer diagnosis, the investigators found an altered prevalence of sepsis in the history of patients with certain cancer types [25]. Because sepsis and septic shock may lead to a long-lasting immune suppressed state, which in other contexts has been associated with an increased risk for cancer, we hypothesized that long-term incidence of cancer may be higher in sepsis survivors than in general population. Because of anticipated detection and reverse causation bias and high 1-year sepsis mortality [3], we focused on 1-year sepsis survivors. The aim of this registry-based study was to assess the incidence of new cancer in 1-year sepsis survivors and compare it with the incidence in the general population. For descriptive purposes, we also calculated the prevalence of malignant diseases in this cohort.

## Methods

### Ethical approval

The study protocol was approved by the Regional ethical board of Uppsala, Sweden ( October 12, 2016, Dnr 2016/421 and 2016-421-1). We follow the STROBE Statement checklist for reporting observational studies in reporting the design and results of this study.

### Data acquisition

This is a registry-based study utilizing data from Swedish Intensive Care Registry (SICR) between January 1st, 2005, and December 31st, 2016, combined with data from National Patient Registry (NPR; Patientregistret), Cancer registry, and Cause of death databases of the National Board of Health and Welfare (Patientregistret, Cancerregistret, Dödsorsaksregistret, Socialstyrelsen). Data from different registries were linked using the unique personal identity number available for every Swedish resident.

SICR (https://www.icuregswe.org/) is a national intensive care quality database collecting data since 2003 from the general ICU in Swedish hospitals (approximately 50,000 admissions annually). SICR did not cover the whole country initially, but the coverage has increased reaching 80 of 84 ICUs in 2017 [26].

Data retrieved from SICR included data on ICU diagnoses recorded using 10^th^ revision of International Classification of Diseases (ICD-10), age at admission, sex, admission and discharge dates, type of admission (emergency or scheduled, operative or non-operative), type of hospital, severity of illness scores (SAPS 3, Simplified Acute Physiology Score 3; APACHE 2, Acute Physiology and Chronic Health Assessment 2), diagnosed cancer with or without metastasis, diagnoses associated with high mortality risk (cirrhosis, cardiac insufficiency, cancer, hematological malignancy, AIDS), ICU length of stay (ICU-LOS), use of organ support, and date of death in the ICU. When registering a case into the SICR, it is mandatory to state whether sepsis is negated. ICU admissions in SICR were considered sepsis-related if at least one of the following diagnoses was registered as an ICU diagnosis: sepsis (ICD-10 A41.9), severe sepsis (R57.2), and septic shock (R65.1) [27].

We also retrieved data from the NPR run by Swedish National Board of Health and Welfare (Socialstyrelsen), that registers all in-patient hospital visits in Sweden. We used diagnosis code data from the NPR to calculate Charlson comorbidity index (CCI) [28].

We sought data on registered cancer diagnoses, and dates of cancer diagnoses from the cancer database of The National Board of Health and Welfare (Cancerregistret, Socialstyrelsen), beginning 6 years before the earliest entry to the study cohort in 2006 and until December 2018. Swedish Cancer Registry (https://ghdx.healthdata.org/record/sweden-cancer-register-2014) database is a high-quality standard registry with complete and accurate data [29]. It includes personal identity number-linked data reported by caregivers. One individual may have several cancer diagnoses in the registry, but each new cancer is registered once. Reporting any malignancy or severe dysplasia, excluding thyroid adenomas and radically excised basal cell carcinomas, is mandatory for all caregivers. A list of benign but life-threatening tumors that are also reported is provided in Additional file 1: Table E1.

By linking the personal identity numbers of the cohort with data from the Cause of death registry (Dödsorsaksregistret, Socialstyrelsen) and NPR, we obtained mortality and emigration data for the whole observation period.

We used general Swedish population as reference population. Data for comparison were retrieved from the NORDCAN database (www.ANCR.nu). NORDCAN project is provided by the Association of Nordic Cancer Registries (ANCR) and has registered cancer incidence, prevalence, and mortality data from Nordic countries since 1943. It is updated annually and is freely available to all users. For calculating the expected (5-years) prevalence rates and incidence rates of all cancers and cancers of individual sites, accounting for sex, age, and year of diagnosis in the 1-year sepsis survivors, we retrieved 5-year prevalence rates and incidence rates for each of the most frequent cancer types for the same time-period in Sweden, according to year, sex, and age in 5-year categories from 20 to > 85 years age from the database. The cancer types that were searched according to the ICD-codes are listed in Additional file 1: Table E2.

### Formation of the study cohort

**Fig. 1:**
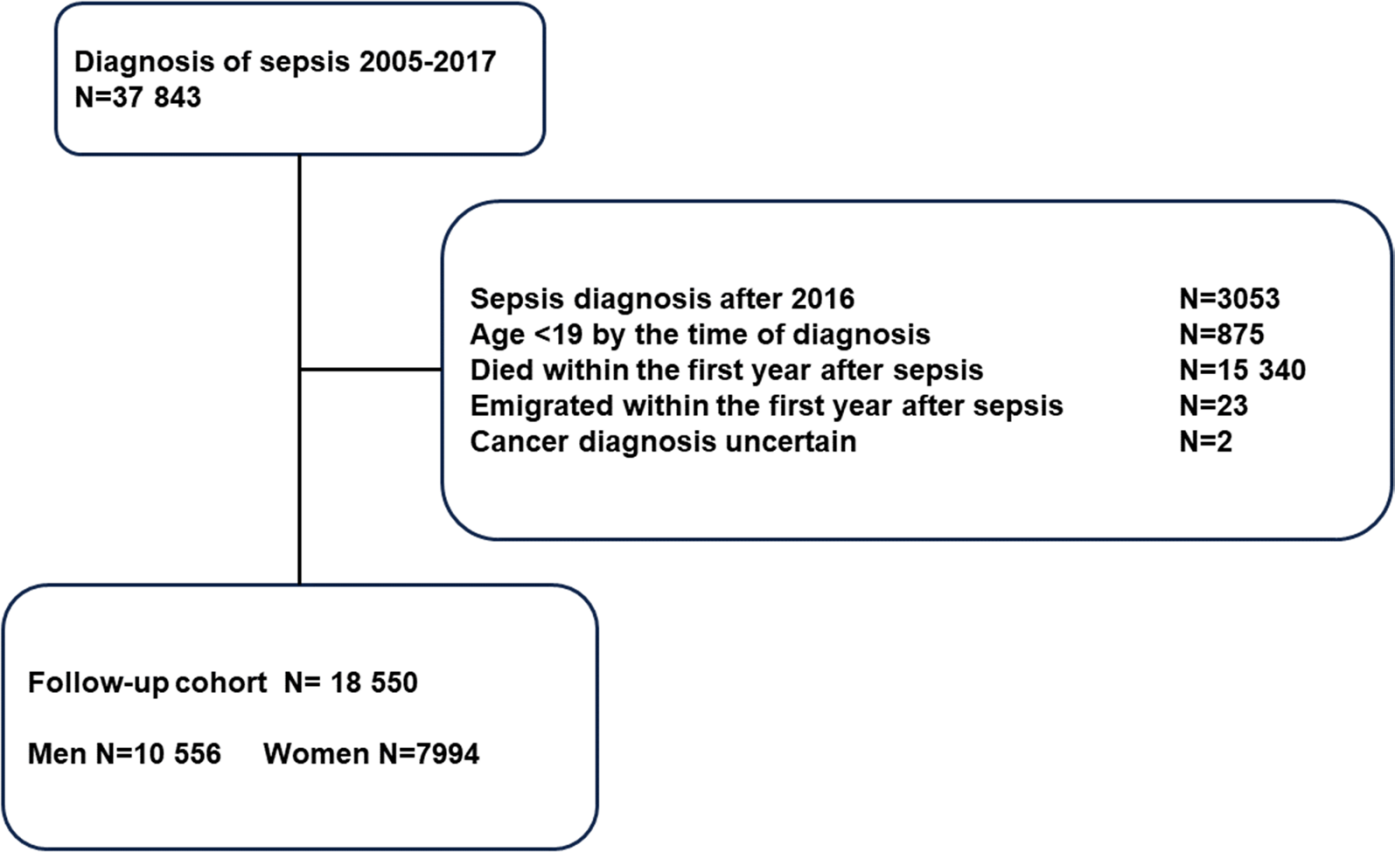
Study flow chart. Study flow chart demonstrates how the final cohort was formed

We identified 37,843 adult (age ≥ 18 years) patients with a sepsis-related ICU admission (excluding readmissions) in SICR between January 1st, 2005, and December 31st, 2017 (Fig. 1). One-year sepsis survivors were used as the study cohort, that was formed by excluding 3053 cases with the index ICU admission in 2017 to allow follow-up for the cancer occurrence for at least 1 year. Due to lack of relevant reference group for 19-year-olds in the NORDCAN database that provides statistics by 5-year age categories, patients 18 years of age at the index ICU admission (age 19 years at entering the cohort) were excluded (*N* = 875). Patients who died (*N* = 15 340) or emigrated (*N* = 23) within the first year after the index admission were excluded from the final cohort.

### Follow-up and end points

The follow-up began 1 year after the date of admission to the ICU (2006–2017) and ended on the date of cancer diagnosis, emigration (*N* = 70), death (*N* = 6366), or December 31st, 2018, whichever came first. The objective of this study was to calculate the incidence rate of any cancer and site-specific cancer whenever reasonable (at least five cases) relative to that of the general population.

### Statistical analysis

We report the results of all analyses separately for women and men. We report continuous variables as medians and inter-quartile ranges (IQR) and categorical variables with numbers and percentages for each category. We report the numbers and percentages of missing values in each category. To describe the frequency of pre-existing cancer diagnosis in the sepsis cohort as compared to the general population, we calculated overall 5-year standardized prevalence ratios (SPR) and 95% confidence intervals (CI). Prevalence ratios were standardized by calendar year and age (5-year categories) at entering the cohort (i.e., 1 year after the index ICU admission) and were calculated as the observed prevalence relative to the expected one using the direct standardization method. The observed prevalence was calculated in each year- and age-specific stratum as the proportion of individuals diagnosed with cancer within 5 years before onset of follow-up of all individuals in this stratum. The corresponding expected prevalence was based on the NORDCAN statistics of prevalence in Swedish population.

We calculated the overall sex-specific as well as sex- and site-specific crude incidence rates (IR) by dividing the total number of cases by the accumulated person-years. We evaluated the differences in the occurrence of cancer in the study population and general Swedish population by means of standardized incidence rate (SIR). SIR is used commonly in cancer epidemiology. It provides a means to calculate a rate that can be expected in a certain population based on a known rate in a larger population, when accounting important factors, such as sex, age, and calendar time. For each of the sex-and site-specific outcomes of interest, we calculated the number of events and person-years by 5-year age categories and year of cancer diagnosis. When calculating the site-specific SIRs, we excluded individuals diagnosed with cancer of interest before the onset of follow-up. We calculated the SIR as the ratio between the observed and the expected number of cases (incidence rate in the Swedish population multiplied by the accumulated person-years in the study population). We calculated IRs and SIRs over the whole follow-up period and by length of follow-up (≤ 1, 1–2, 3–4, and ≥ 5 years). We calculated 95% CI for the IR and SIR by assuming Poisson distribution of the observed cases.

We performed several sensitivity analyses. First, previous cancer may be associated with a higher risk of new cancer. Therefore, imbalance in the proportion of individuals with history of cancer between 1-year sepsis survivors and general population is likely to affect the SIR. Being unable to adjust for the imbalance, we calculated SIRs according to history of cancer. Second, we excluded non-melanoma of the skin from the analysis of any cancer, because this most common cancer type tends to be detected and/or reported with frequency varying between the population subgroups. Third, we performed the analyses by age at cancer diagnosis to study whether the relative difference in the incidence of any cancer was affected by age. Fourth, expecting a likely accumulation of shared risk factors of cancer and sepsis in patients with comorbidities, we performed the analyses according to CCI.

## Results

The final cohort comprised 18,550 1-year sepsis survivors; 10,556 (56.9%) were men, almost half (45%) entered the cohort in 2014–2017 (ICU admission 2013–2016) and were predominately of late middle age or elderly (median 66, IQR 55–75 years) at entry. In great majority of men (85.5%) and women (91%), CCI was 0 (Table 1).

**Table 1:** Characteristics of the cohort of one-year sepsis survivors

	Men *N* = 10 556	Women *N* = 7994
*Admission year, N (%)*		
2005–2008	2074 (19.6)	1557 (19.5)
2009–2012	3721 (35.3)	2773 (34.7)
2013–2016	4761 (45.1)	3664 (45.8)
Age at sepsis diagnosis, median (IQR)	67 (56–75)	65 (52–74)
*Charlson comorbidity index, N (%)*		
0	9 021 (85.5)	7 276 (91.0)
1–2	669 (6.3)	355 (4.4)
3 or more	849 (8)	344 (4.3)
Data missing	17 (0.2)	19 (0.2)
*AIDS, N (%)*		
Yes	11 (0.1)	6 (0.1)
No	8319 (78.8)	6350 (79.4)
Data missing	2226 (21.1)	1638 (20.5)
*Cirrhosis, N (%)*		
Yes	112 (1.1)	86 (1.1)
No	8218 (77.9)	6270 (78.4)
Data missing	2226 (21.1)	1638 (20.5)
*Admission type*		
Surgical admission, *N* (%)	1523 (14.4)	1304 (16.3)
Elective, *N* (%)	281 (18.5)	193 (14.8)
Emergency, *N* (%)	1242 (81.5)	1111 (85.2)
Data missing	53 (0.5)	19 (0.24)
*Emergency admission, N (%)*		
Yes	10,041 (95.5)	7683 (96.1)
No	477 (4.2)	298 (3.7)
Data missing	38 (0.3)	13 (0.2)
SAPS 3, median (IQR)	62 (54–71)	60 (52–69)
*Vasoactive treatment on admission, No (%)*		
Yes	1330 (12.6)	1030 (12.9)
No	7000 (66.3)	5326 (66.6)
Data missing	2223 (21.1)	1638 (20.5)
*ICU length of stay, N (%)*		
< 1 d	4446 (42.1)	3336 (41.7)
1–2 d	2077 (19.7)	1740 (21.8)
3–7 d	1025 (9.7)	741 (9.3)
8–14 d	391 (3.7)	273 (3.4)
> 14 d	391 (3.7)	266 (3.3)
Data missing	2223 (21.1)	1638 (20.5)
Cancer diagnosis within 5 years before onset of follow-up*, N (%)		
Yes	2324 (22)	1384 (17.3)
No	8232 (78)	6610 (82.7)
*Ongoing cancer treatment on admission, N (%)*		
Yes	657 (6.2)	590 (7.4)
No	7673 (72.7)	5766 (72.1)
Data missing	2226 (21.1)	1638 (20.5)

Characteristics of the cohort of one-year sepsis survivors at the index ICU admission or at the start of follow-up (i.e., one year after the index admission). *IQR* inter-quartile range, *SAPS 3* Simplified Acute Physiology Score 3, *AIDS* acquired immune deficiency syndrome

*Based on data from Swedish Cancer Registry

Previous cancer diagnoses within 5 years before the onset of the follow-up were found for 3708 (20.0%) patients, 1247 (6.7%) patients had ongoing treatment for cancer by time of ICU admission. New cancer was diagnosed in 550 (3.0%) patients during the first year after the index admission (before the onset of follow-up). The SPRs for all cancers for the 5 years before onset of follow-up were 4.46 (95% CI 4.28–4.64) in men and 5.40 (95% CI 5.12–5.69) in women.

When followed up for any new cancer, 75,427 person-years (41,710 for men, 33,718 for women) accumulated and 1625 (8.8%) were diagnosed with at least one new cancer during a median follow-up of 3.36 (IQR 1.72–5.86) years. Number of new cancer diagnoses during follow-up was 1041 for men and 692 for women. Median time to the first diagnosis of any new cancer was 2.51 (IQR 1.09–4.48) years. The SIRs with 95% CI of any new cancer are shown in Table 2.

**Table 2:** Follow-up data of the study cohort

	Men, *N* = 10 556	Women, *N* = 7994
Duration of follow-up (years), median (IQR)	3.22 (1.64–5.71)	3.56 (1.82–6.08)
Person-years	41,710.2	33,717.6
Diagnosed with new cancer, *N* (%)	967 (9.2)	658 (8.2)
Time to first cancer diagnosis, years, median (IQR)	2.54 (1.05–4.54)	2.33 (1.08–4.24)
Age at time of new cancer diagnosis, years, median (IQR)	72.9 (66.5–79.4)	71.0 (62.6–78.5)
Death during follow-up, *N* (%)	3888 (34.3)	2478 (30.1)
Any cancer, SIR (95% CI), whole follow-up Whole cohort	1.31 (1.23–1.40)	1.74 (1.61–1.88)
Any cancer, SIR (95% CI) by follow-up period, whole cohort		
< 1 year	2.13 (1.87–2.42)	2.88 (2.45–3.38)
1–2 years	1.16 (1.05–1.30)	1.67 (1.47–1.90)
3–4 years	1.20 (1.05–1.38)	1.69 (1.44–1.99)
≥ 5 years	1.16 (1.01–1.33)	1.25 (1.05–1.50)

New diagnoses of any cancer and mortality during follow-up, according to sex are shown

*PY* person years, *IQR* inter-quartile range, *SIR* standardized incidence ratio

A roughly twofold incidence of any cancer was observed during the first year of follow-up in both men and women compared with the general population. The magnitude decreased thereafter for both sexes. However, the number of observed cancer cases remained higher than expected, regardless age (SIR was highest in the youngest age group < 40 years of age), CCI, and when excluding patients with previous cancer diagnosis and non-melanoma of the skin (Table 3). In men without history of cancer, the incidence was no longer different from that of the general population after 3 years of follow-up. As the acuity of disease was low to moderate in the cohort of 1-year survivors, we performed an additional analysis calculating SIR in three categories according to SAPS 3 score. The number of observed remained higher than expected regardless SAPS 3 category. Values were missing in 21%. (Additional file 1: Table E3).

**Table 3:** Results from the sensitivity analyses

Any cancer, SIR (95% CI)	Men	Women
*History of cancer*		
Yes	1.31 (1.16–1.48)	1.67 (1.41–1.98)
No	1.32 (1.22–1.42)	1.76 (1.61–1.91)
*Without history of cancer, by length of follow-up*		
< 1 year	1.89 (1.59–2.24)	2.72 (2.23–3.31)
1–2 years	1.14 (1.00–1.31)	1.57 (1.34–1.83)
3–4 years	1.00 (0.83–1.20)	1.52 (1.25–1.85)
≥ 5 years	1.12 (0.85–1.32)	1.29 (1.06–1.58)
Non-melanoma of the skin excluded (all patients included)	1.18 (1.10–1.27)	1.59 (1.46–1.73)
*Age (all patients included)*		
< 40	4.96 (2.75–8.96)	5.94 (4.10–8.60)
40–49	2.60 (1.59–4.25)	2.96 (2.15–4.09)
50–59	2.19 (1.75–2.75)	2.38 (1.88–3.20)
60–69	1.40 (1.24–1.58)	1.89 (1.63–2.19)
70–79	1.20 (1.08–1.32)	1.52 (1.33–1.74)
≥ 80	1.19 (1.04–1.35)	1.38 (1.17–1.63)
*Charlson comorbidity index (all patients included)*		
0	1.30 (1.21–1.39)	1.75 (1.62–1.90)
1–2	1.47 (1.18–1.84)	1.67 (1.19–2.36)
≥ 3	1.35 (1.10–1.68)	1.60 (1.10–2.34)

Patients with previous cancer diagnosis, new cancers excluding non-melanoma of the skin, new cancers in different age categories (year of cancer diagnosis) and according to comorbidities

*SIR* standardized incidence ratio, *95% CI* 95% confidence interval

**Fig. 2:**
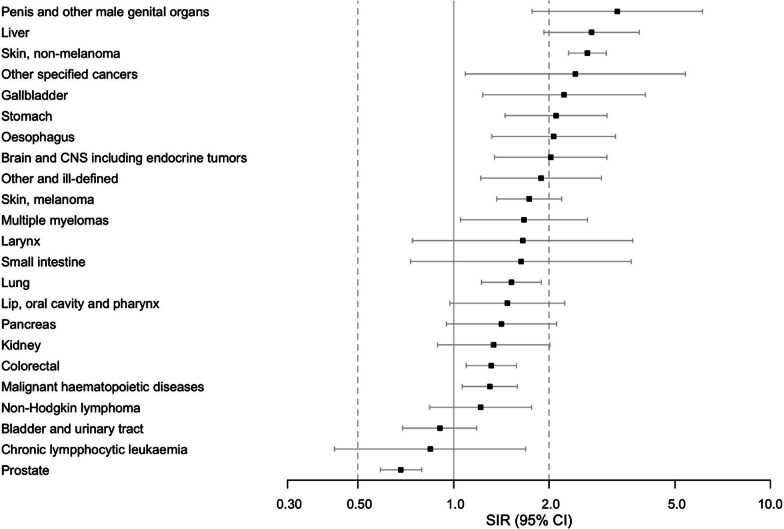
Standardized incidence rates of individual cancers in men. Forest plot presents standardized incidence ratios (SIR) (ratio of observed and expected incidence) for individual cancers with 95% CI: s throughout the study period for men surviving at least one year after sepsis. Cancer sites with at least five cases are presented

Site-specific SIRs and 95% CIs over the whole study period are shown in Figs. 2 (men) and 3 (women).

**Fig. 3:**
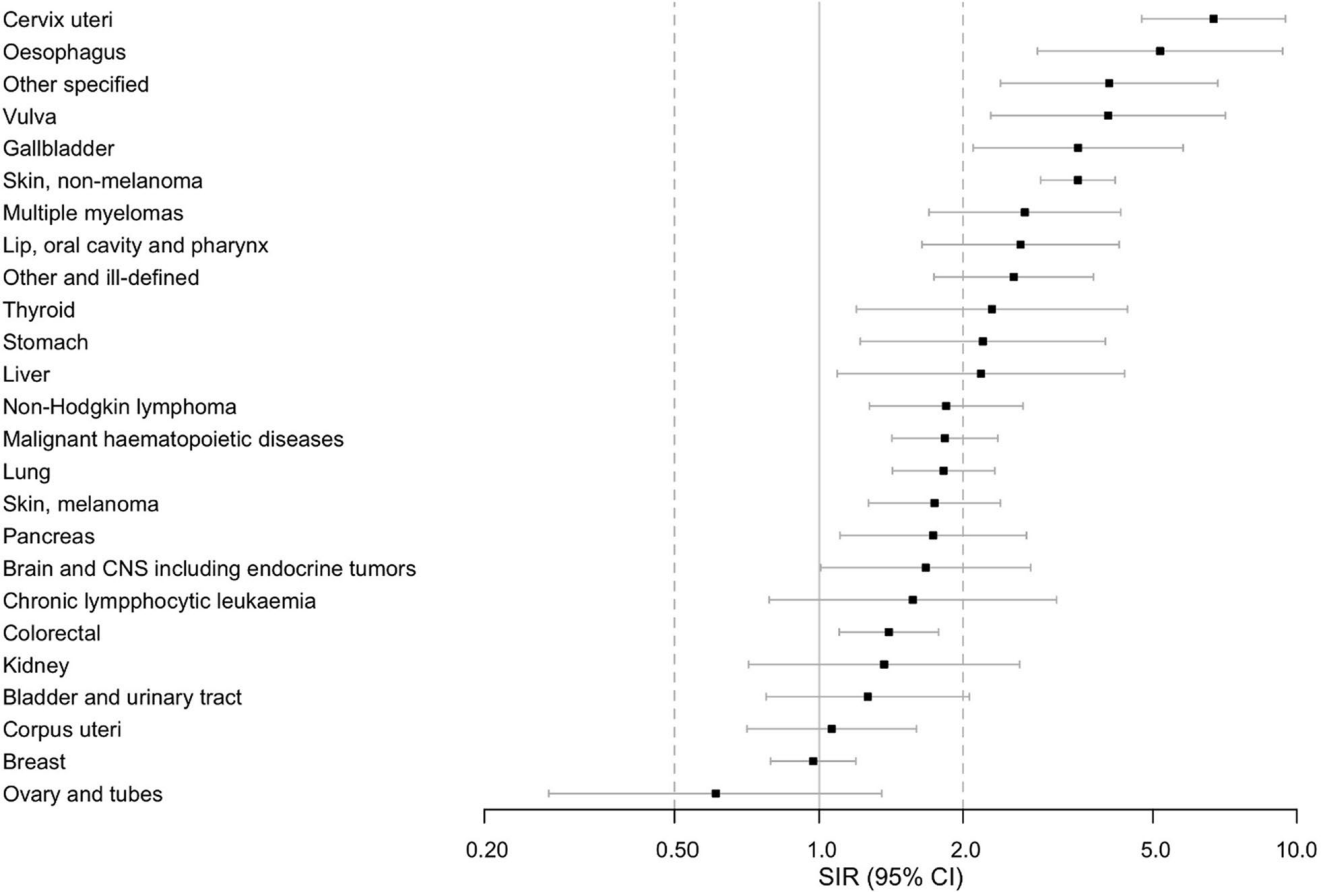
Standardized incidence rates of individual cancers in women. Forest plot presents standardized incidence ratios (SIR) (ratio of observed and expected incidence) for individual cancers with 95% CI: s throughout the study period for women surviving at least one year after sepsis. Cancer sites with at least five cases are presented

In both sexes, we observed an increased incidence of cancers of the skin, gastro-intestinal tract (esophagus, stomach, and colon/rectum), gallbladder, liver, lung, brain, and malignant hematopoietic diseases including multiple myeloma, and a group of other, ill-defined cancers. We observed an increased incidence of cancers of the reproductive organs such as cervix and vulva in women and penile cancer in men, and a lower incidence of prostatic cancer. Considering length of follow-up (< 1, 1–2, 3–4 or > 5 years), only the SIR for non-melanoma of the skin in both sexes and cervical cancer in women was statistically significantly increased regardless the length of follow-up. Detailed data on sex and site-specific SIRs, including the number of cancer cases and crude and adjusted incidence rates (IR), are available in Additional file 1: Tables E4–E7.

## Discussion

In this nationwide registry-based study, we found that 1-year sepsis survivors have a higher incidence of cancer than the general population. This was observed in both sexes and remained when patients with previous cancer diagnosis and cases with the most frequent new cancer, non-melanoma of the skin, were excluded. SIRs were elevated for cancers of the skin, gastro-intestinal tract, gallbladder, liver, lung, central nervous system, reproductive organs, malignant hematological diseases, and a group of ill-defined cancers. The SIR was highest during the first year of follow-up abating thereafter. Concerning individual cancers, the SIR remained elevated during the whole follow-up period only for non-melanoma of the skin in both sexes and cervical cancer in women. Possible explanations to the temporally decreasing SIR include increased detection rate after the index hospitalization, reverse causation, or death as a competing event in this population with a considerably high mortality.

Strengths of our study include a large population with a nearly complete follow-up data, based on reliable registries. We included adult patients of all ages and calculated SIR according to age. We performed several sensitivity analyses to test the robustness of the results. To our knowledge, the incidence of malignant disease in a large cohort of sepsis patients and considering FU time has not been reported before.

There are limitations in our study. First, the diagnose codes we used are explicit for sepsis, severe sepsis, or septic shock, dating before the current sepsis definition (Sepsis-3) [30], and some misclassification likely exists [3, 31]. Second, we included only patients treated in ICUs. These weaknesses limit the generalizability of our results to sepsis defined by the current definition and sepsis treated in non-ICU settings. Third, shared risk factors for sepsis and cancer such as comorbidities and life-style-related factors, e. g., smoking and obesity, likely accumulate in this population [32–34]. Unfortunately, reliable data on important life-style factors were not available, neither could we account for comorbidity in the reference population. Majority in our cohort had no comorbidities and the severity of acute disease was low to moderate, suggesting selection through mortality during the first post-admission year. Fourth, the time frame of the study may cause underestimation of the importance of prior cancer and underestimation of cancers with slow progression [35–37]. Fifth, high post-sepsis mortality is a competing risk, limits the length of follow-up and introduces selection bias. Sixth, despite focusing in 1-year survivors, detection bias and reverse causation cannot be excluded. We observed a U-shaped temporal variation of SIR in certain cancers, which suggests a possible detection bias that pushed toward earlier diagnoses. Seventh, surveillance bias cannot be excluded, as the cancer types with most consistently elevated SIRs were non-melanoma of the skin and cervical cancer, suggesting a role of diagnostic activity. Finally, our study design and results do not infer causality between sepsis and cancer.

A recent study in elderly found more sepsis in the history of patients with first diagnosis of cancer than in matched controls [25]. We included also younger patients and, interestingly, observed highest SIRs in the younger age groups. Concerning cancer sites, the findings of Liu et al. were in broad accordance with our findings and those reported for solid organ transplantation patients [23, 25]. They found inverse associations for several cancers, including cancer of the prostate, that was also in our study less frequent in sepsis survivors [25]. However, important differences in the approaches between their study and ours exist, with different sources of potential selection bias. For example, as their design allowed including only patients surviving until cancer diagnosis, mortality in our cohort was a competing event to cancer diagnosis. Indeed, high mortality may partly explain the declining power of our findings in longer follow-up.

Future studies including patients with sepsis according to Sepsis-3 definition, with a prospective design, longer follow-up and focus on lifestyle factors and other common risk factors are needed to examine the association of sepsis and cancer. The age-standardized incidence rates and cumulative risk of all cancer for adults in Sweden are close to the median rates in Europe, which are higher than those in Africa, Asia, and Latin America, but lower than in North America and Oceania [38]. Repeating the result in other populations is warranted to confirm our findings. However, according to results by others and this study, surveillance for malignant disease may be warranted in sepsis survivors.

## Conclusions

Compared to general population, incidence of new cancer was increased in one-year survivors of ICU-treated sepsis. Variation in the findings depending on follow-up time suggests that factors other than sepsis alone are involved.

### Supplementary Information

**Additional file 1**. **Table E1**: Benign tumors that are reported to Cancer registry. **Table E2**: Cancer types according to ICD–codes. **Table E3**: Standardized incidence ratio of new cancer in men and women according to severity of acute disease. **Table E4**: Site-specific a) standardized incidence ratios (SIR) and b) crude and adjusted incidence rates (IR) of cancer per 100 000 person years for men. **Table E5**: Site-specific a) standardized incidence ratios (SIR) and b) crude and adjusted incidence rates (IR) of cancer per 100,000 person years for women. **Table E6**: Site-specific standardized incidence ratios (SIR) of cancer by length of follow up for men. **Table E7**: Site-specific standardized incidence ratios (SIR) of cancer by length of follow-up for women.

## Data Availability

The data used in this study are available in the Swedish Intensive Care Registry, National patient registry, Cancer registry and Cause of death registry. The data were used under licence for the current study and thus are not publicly available. The data, however, are available from the registries upon reasonable request after ethical review and with permission of the Swedish Intensive Care Registry and the Swedish National Board of Health and Welfare.

## Abbreviations

AIDS: Acquired immune deficiency syndrome
ANCR: Association of Nordic Cancer Registries
APACHE 2: Acute Physiology and Chronic Health Assessment 2
CCI: Charlson comorbidity index
CI: Confidence interval
FU: Follow-up
HIV: Human immune deficiency virus
ICD-10: International Classification of Diseases-10
ICU: Intensive care unit
ICU-LOS: Intensive care unit length-of-stay
IQR: Inter-quartile range
IR: Incidence rate
NPR: National Patient Registry
SAPS 3: Simplified Acute Physiology Score 3
SICR: Swedish Intensive Care Registry
SIR: Standardized incidence ratio
SPR: Standardized prevalence ratio
STROBE: Strengthening the Reporting of Observational and Epidemiological studies
