# Supplementary material for: Risk of malignant disease in 1-year sepsis survivors, a registry-based nationwide follow-up study
Source: Crit Care. 2023 Sep 29;27:376. doi: 10.1186/s13054-023-04654-9 (PMC10543324; doi:10.1186/s13054-023-04654-9)
Supplement: Supplementary file 1 — Additional file 1. Table E1: Benign tumors that are reported to Cancer registry. Table E2: Cancer types according to ICD–codes. Table E3: Standardized incidence ratio of new cancer in men and women according to severity of acute disease. Table E4: Site-specific a) standardized incidence ratios (SIR) and b) crude and adjusted incidence rates (IR) of cancer per 100 000 person years for men. Table E5: Site-specific a) standardized incidence ratios (SIR) and b) crude and adjusted incidence rates (IR) of cancer per 100,000 person years for women. Table E6: Site-specific standardized incidence ratios (SIR) of cancer by length of follow up for men. Table E7: Site-specific standardized incidence ratios (SIR) of cancer by length of follow-up for women. [file 13054_2023_4654_MOESM1_ESM.pdf]

Additional material

INDEX

TABLE E 1. Benign tumors that are reported to Cancer registry 2

TABLE E 2. Cancer types according to ICD–codes 3

TABLE E 3. Standardized incidence ratio of new cancer in men and women according to severity of acute disease. 4

TABLE E 4. Site–specific a) standardized incidence ratios (SIR) and b) crude and adjusted incidence rates (IR) of cancer per 100 000 person years for men 5

TABLE E 5. Site–specific a) standardized incidence ratios (SIR) and b) crude and adjusted incidence rates (IR) of cancer per 100 000 person years for women 7

TABLE E 6. Site–specific standardized incidence ratios (SIR) of cancer by length of follow up for men 9

TABLE E 7. Site–specific standardized incidence ratios (SIR) of cancer by length of follow up for women 11

TABLE E 1. Benign tumors that are reported to Cancer registry.

Thymus	Thymoma
Endocrine glands	All diagnoses except thyroid adenoma and adenoma of the adrenal cortex with no endocrine activity
Placenta	Complete or partial mola hydatidosa and trophoblast tumors originating in placenta
Nasal cavity	Schneider tumor
Intracranial or intraspinal	All diagnoses
Testicles	Teratomas, Leydig and Sertoli cell tumors, Sertoli–Leydig cell tumors
Urinary bladder and urinary system	Urothelial papilloma except inverted papilloma and papilloma without atypia
Ovary	Borderline tumors, theca cell tumors, granuloma–theca cell tumors, luteoma, Leydig and Sertoli cell tumors, Sertoli–Leydig cell tumors
Orbit	Opticus nerve meningeoma
Nasopharynx	Juvenile angiofibroma

TABLE E 2. Cancer types according to ICD–codes

Cancer types
Hemato.lymph="^C8[1–6]|^C88|^C9[0–6]|^D45|^D46|^D47[0–1]|^D47[3–9]",<br>Colorectal.cancer="^C18|^C19|^C20|^C21",<br>Lung.cancer="^C33|^C34",<br>Skin.nonmelanoma="^C44|^C460",<br>Prostate.cancer="^C61",<br>Pancreatic.cancer="^C25|^157",<br>Bladder.cancer="^C6[5–8]|^D09[0–1]|^D30[1–9]|^D41[1–9]",<br>Liver.cancer="^C22",<br>Gallbladder.cancer="^C2[3–4]",<br>Stomach="^C16",<br>Kidney.cancer="^C64",<br>Skin.melanoma="^C43",<br>Breast.cancer="^C50",<br>Small.intestine="^C17",<br>Brain.nervs="^C7[0–2]|^C75[1–3]|^D3[2–3]|^D35[2–4]|^D4[2–3]|^D44[3–5]",<br>Oesophagus="^C15",<br>Uterus.cancer="^C54",<br>Ovary.cancer="^C56|^57[0–4]",<br>Cervix.cancer="^C53",<br>Lip.cancer="^C00[0–2]|^C00[5–9]",<br>Oropharynx.cancer="^C01|^C05[1–9]|^C09|^C100|^C10[2–9]",<br>Oral.cavity="C00[3–4]|^C0[2–4]|^C050|^C06",<br>Pharynx="^C0[7–8]",<br>Larynx="^C32|^C101",<br>Hypopharynx="^C1[2–3]",<br>Soft.tissue="^C49|^C461",<br>Thyroid.cancer="^C73",<br>Testis.cancer="^C62",<br>Uterus.unspec="^C55|^C58",<br>Other.gynorg.w="^C5[1–2]|^C57[7–9]",<br>Other.genital.org.m="^C60|^C63",<br>Pleura="^C384|^C450|^C45.9",<br>Bone="^C4[0–1]",<br>Nasal="^C3[0–1]",<br>Other.spec="^C37|^C38[0–3]|^C388|^C45[1–7]|^C46[2–9]|^C4[7–8]|^C74|^C750|^C75[4–9]",<br>Unknown.unspec="^C26|^C39|^C7[6–9]|^C80|^C97"

Table E 3. Standardized incidence ratio of new cancer in men and women according to

severity of acute disease. The cohort was divided in three categories of equal size based on admission SAPS III scores. SAPS III values were missing in 21 %.

**Men**	**SAPS available, N**<br>**data missing**	**8292 (78.5)**<br>**2264 (21.4)**		
	Median (IQR)	62 (54–71)		
	Mean (SD)	62.77 (12.57)		
	SAPS	N new cancer	SIR	95 % CI
	<57	192	1.49	1.28–1.70
	57-<67	198	1.13	0.98–1.29
	≥67	250	1.25	1.10–1.41
**Women**	**SAPS available, N**<br>**data missing**	**6323 (79.1)**<br>**1671 (20.9)**		
	Median (IQR)	60 (52–69)		
	Mean (SD)	61.01 (12.62)		
	SAPS	N new cancer	SIR	95 % CI
	<55	115	2.03	1.69–2.43
	55-<66	168	1.71	1.47–1.99
	≥66	170	1.62	1.39–1.88

SAPS III= Simplified Acute Physiology Score III, IQR= Inter-Quartile Range, SD= Standard deviation, 95 % CI= 95 % confidence interval

TABLE E 4. Site–specific a) standardized incidence ratios (SIR) and b) crude and adjusted incidence rates (IR) of cancer per 100 000 person years for men. SIR and IR are presented with lower (lCI95) and upper (uCI95) limits of 95% confidence intervals. Cancers with >15 cases are shown.

a)

Cancer, site	N observed	N expected	SIR	lCI95	uCI95
Skin_nonmelanoma	204	77.18	2.64	2.30	3.03
Prostate	176	257.64	0.68	0.59	0.79
Colorectal	115	87.50	1.31	1.09	1.58
Malignant_hematopoietic	95	73.09	1.30	1.06	1.59
Lung	81	53.23	1.52	1.22	1.89
Skin_melanoma	69	39.85	1.73	1.37	2.19
Urinary bladder	54	59.70	0.90	0.69	1.18
Liver	32	11.75	2.72	1.93	3.85
Stomach	28	13.30	2.10	1.45	3.05
Non-Hodgkin_lymphoma	28	23.02	1.22	0.84	1.76
Pancreas	24	16.97	1.41	0.95	2.11
Kidney	23	17.21	1.34	0.89	2.01
Brain_CNS_including_endocrine	23	11.37	2.02	1.34	3.05
Lip_oral_cavity_pharynx	22	14.90	1.48	0.97	2.24
Other_illdefined	20	10.60	1.89	1.22	2.93
Oesophagus	19	9.19	2.07	1.32	3.24
Multiple_myelomas	18	10.80	1.67	1.05	2.65
Gallbladder	11	4.93	2.23	1.23	4.03
Penis_other	10	3.04	3.28	1.77	6.10
Chronic_lymphatic_leukemia	8	9.48	0.84	0.42	1.69
Small_intestine	6	3.67	1.63	0.73	3.63
Larynx	6	3.63	1.65	0.74	3.68
Other_specified	6	2.48	2.42	1.09	5.39
Thyroid	5	2.52	1.98	0.83	4.77
Soft_tissue	5	3.72	1.34	0.56	3.23
Acute_myeloid_leukemia	5	3.50	1.43	0.59	3.43
Chronic_myeloid_leukemia	4	2.06	1.94	0.73	5.17
Pleura	3	2.68	1.12	0.36	3.47
Nasal_cavity_middle_ear_sinuses	2	0.95	2.10	0.53	8.42
Testis	1	1.76	0.57	0.08	4.03
Eye	1	1.50	0.67	0.09	4.75
Acute_lymphatic_leukemia	1	0.34	2.95	0.42	20.92
Bone	0	0.66	0.00	0.00	

CNS, central nervous system

b)

Cancer, site	Crude rate	Adjusted rate	lCI95	uCI95
Skin_nonmelanoma	471.32	330.77	288.35	379.42
Prostate	405.99	256.22	221.03	297.01
Colorectal	263.90	178.99	149.10	214.89
Malignant_hematopoietic	217.74	133.84	109.46	163.65
Lung	185.19	118.25	95.11	147.03
Skin_melanoma	158.14	102.71	81.12	130.04
Urinary bladder	123.62	81.74	62.60	106.73
Liver	73.13	42.38	29.97	59.93
Non-Hodgkin_lymphoma	63.99	43.41	29.97	62.87
Stomach	63.97	47.57	32.84	68.89
Pancreas	54.80	31.04	20.80	46.31
Brain_CNS_including_endocrine	52.59	37.24	24.75	56.04
Kidney	52.56	31.97	21.24	48.10
Lip_oral_cavity_pharynx	50.26	32.82	21.61	49.85
Other_illdefined	45.68	37.69	24.32	58.42
Oesophagus	43.40	27.29	17.40	42.78
Multiple_myelomas	41.12	26.29	16.57	41.73
Gallbladder	25.11	15.23	8.43	27.50
Penis_other	22.84	14.59	7.85	27.12
Chronic_lymphatic_leukemia	18.27	12.02	6.01	24.04
Small_intestine	13.70	9.34	4.20	20.79
Larynx	13.70	9.12	4.10	20.30
Other_specified	13.70	9.77	4.39	21.75
Thyroid	11.42	8.21	3.42	19.72
Soft_tissue	11.42	8.41	3.50	20.20
Acute_myeloid_leukemia	11.41	8.66	3.60	20.80
Chronic_myeloid_leukemia	9.13	6.65	2.50	17.73
Pleura	6.85	4.43	1.43	13.73
Nasal_cavity_middle_ear_sinuses	4.57	3.28	0.82	13.12
Testis	2.28	4.30	0.61	30.55
Eye	2.28	1.56	0.22	11.07
Acute_lymphatic_leukemia	2.28	2.23	0.31	15.81
Bone	0.00	0.00	0.00	0.00

CNS, central nervous system

TABLE E 5. Site–specific a) standardized incidence ratios (SIR) and b) crude and adjusted incidence rates (IR) of cancer per 100 000 person years for women. SIR and IR are presented with lower (lCI95) and upper (uCI95) limits of 95% confidence intervals. Cancers with >15 cases are shown.

a)

Cancer, site	N observed	N expected	SIR	lCI95	uCI95
Skin_nonmelanoma	119	34.19	3.48	2.91	4.17
Breast	91	93.67	0.97	0.79	1.19
Colorectal	67	47.88	1.40	1.10	1.78
Lung	63	34.59	1.82	1.42	2.33
Malignant_hematopoietic	59	32.19	1.83	1.42	2.37
Skin_melanoma	38	21.80	1.74	1.27	2.40
Cervix_uteri	32	4.78	6.69	4.73	9.47
Non-Hodgkin_lymphoma	28	15.18	1.84	1.27	2.67
Other_illdefined	26	10.18	2.56	1.74	3.75
Corpus_uteri	23	21.65	1.06	0.71	1.60
Pancreas	19	10.97	1.73	1.11	2.72
Multiple_myelomas	18	6.68	2.70	1.70	4.28
Lip_oral_cavity_pharynx	17	6.44	2.64	1.64	4.25
Bladder_urinary	16	12.66	1.26	0.77	2.06
Gallbladder	15	4.31	3.48	2.10	5.78
Brain_CNS_including_endocrine	15	8.97	1.67	1.01	2.77
Other_specified	14	3.46	4.05	2.40	6.83
Vulva	12	2.98	4.03	2.29	7.09
Oesophagus	11	2.13	5.17	2.86	9.34
Stomach	11	5.00	2.20	1.22	3.97
Kidney	9	6.58	1.37	0.71	2.63
Thyroid	9	3.91	2.30	1.20	4.42
Liver	8	3.67	2.18	1.09	4.36
Chronic_lymphatic_leukemia	8	5.09	1.57	0.79	3.14
Ovary_tubes	6	9.88	0.61	0.27	1.35
Acute_myeloid_leukemia	5	2.85	1.75	0.73	4.21
Small_intestine	4	1.93	2.07	0.78	5.53
Chronic_myeloid_leukemia	4	1.26	3.18	1.19	8.46
Soft_tissue	2	1.66	1.21	0.30	4.82
Vagina	1	0.49	2.04	0.29	14.51
Uterus_other	1	2.02	0.49	0.07	3.51
Larynx	1	0.46	2.19	0.31	15.57
Nasal_cavity_middle_ear_sinuses	1	0.45	2.22	0.31	15.77
Eye	1	0.84	1.20	0.17	8.49
Acute_lymphatic_leukemia	1	0.33	3.02	0.43	21.46
Pleura	0	0.31	0.00	0.00	
Bone	0	0.39	0.00	0.00	

CNS, central nervous system

b)

**Cancer, site**	**Crude rate**	**Adjusted rate**	**lCI95**	**uCI95**
Skin_nonmelanoma	341.39	255.03	213.09	305.22
Breast	260.89	198.60	161.72	243.90
Colorectal	191.24	140.78	110.81	178.87
Lung	179.49	116.22	90.79	148.77
Malignant_hematopoietic	168.32	122.33	94.78	157.89
Skin_melanoma	108.32	81.18	59.07	111.57
Cervix_uteri	91.20	89.73	63.45	126.88
Other_illdefined	73.94	62.79	42.75	92.22
Corpus_uteri	65.53	47.58	31.62	71.60
Non-Hodgkin_lymphoma	63.99	43.53	30.06	63.05
Pancreas	54.05	34.14	21.77	53.52
Lip_oral_cavity_pharynx	48.40	34.81	21.64	56.00
Bladder_urinary	45.52	32.00	19.60	52.23
Brain_CNS_including_endocrine	42.71	33.17	20.00	55.02
Gallbladder	42.67	29.34	17.69	48.67
Multiple_myelomas	41.12	27.46	17.30	43.59
Other_specified	39.86	27.52	16.30	46.47
Vulva	34.15	25.80	14.65	45.43
Stomach	31.29	24.98	13.83	45.10
Oesophagus	31.28	21.89	12.12	39.52
Thyroid	25.61	21.87	11.38	42.04
Kidney	25.60	17.88	9.30	34.36
Liver	22.76	15.18	7.59	30.36
Chronic_lymph_leukemia	18.27	11.58	5.79	23.16
Ovary_tubes	17.07	13.13	5.90	29.22
Acute_myeloid_leukemia	11.41	8.43	3.51	20.25
Small_intestine	11.38	8.29	3.11	22.08
Chronic_myeloid_leukemia	9.13	6.47	2.43	17.25
Soft_tissue	5.69	4.76	1.19	19.05
Vagina	2.84	2.28	0.32	16.18
Uterus_other	2.84	1.94	0.27	13.76
Larynx	2.84	2.12	0.30	15.07
Nasal_cavity_middle_ear_sinuses	2.84	2.17	0.31	15.37
Eye	2.84	2.11	0.30	15.00
Acute_lymph_leukemia	2.28	1.78	0.25	12.66
Pleura	0.00	0.00	0.00	0.00
Bone	0.00	0.00	0.00	0.00

CNS, central nervous system

TABLE E 6. Site–specific standardized incidence ratios (SIR) of cancer by length of follow up for men. SIRs are presented with lower (lCI95) and upper (uCI95) limits of 95% confidence intervals. Cancers with >15 cases are shown.

Cancer. site	Follow–up time, years	N observed	N expected	SIR	lCI95	uCI95
Urinary bladder	<1	17	8.14	2.09	1.30	3.36
Urinary bladder	1–2	20	22.51	0.89	0.57	1.38
Urinary bladder	3–4	7	13.89	0.50	0.24	1.06
Urinary bladder	>=5	10	15.16	0.66	0.35	1.23
Brain_CNS_incl_endoc	<1	8	1.63	4.92	2.46	9.84
Brain_CNS_incl_endoc	1–2	5	4.30	1.16	0.48	2.79
Brain_CNS_incl_endoc	3–4	6	2.66	2.25	1.01	5.02
Brain_CNS_incl_endoc	>=5	4	2.78	1.44	0.54	3.84
Colorectal	<1	19	12.28	1.55	0.99	2.43
Colorectal	1–2	36	33.23	1.08	0.78	1.50
Colorectal	3–4	32	20.24	1.58	1.12	2.24
Colorectal	>=5	28	21.75	1.29	0.89	1.86
Kidney	<1	5	2.38	2.10	0.87	5.05
Kidney	1–2	7	6.45	1.09	0.52	2.28
Kidney	3–4	1	4.01	0.25	0.04	1.77
Kidney	>=5	10	4.37	2.29	1.23	4.25
Lip_oral_cavity_pharynx	<1	4	2.06	1.94	0.73	5.18
Lip_oral_cavity_pharynx	1–2	13	5.60	2.32	1.35	4.00
Lip_oral_cavity_pharynx	3–4	3	3.49	0.86	0.28	2.67
Lip_oral_cavity_pharynx	>=5	2	3.76	0.53	0.13	2.13
Liver	<1	9	1.54	5.85	3.04	11.23
Liver	1–2	9	4.35	2.07	1.08	3.98
Liver	3–4	5	2.77	1.81	0.75	4.34
Liver	>=5	9	3.09	2.91	1.51	5.60
Lung	<1	18	7.59	2.37	1.49	3.76
Lung	1–2	37	20.31	1.82	1.32	2.51
Lung	3–4	12	12.35	0.97	0.55	1.71
Lung	>=5	14	12.97	1.08	0.64	1.82
Malignant_hematopoietic	<1	23	10.26	2.24	1.49	3.37
Malignant_hematopoietic	1–2	30	27.57	1.09	0.76	1.56
Malignant_hematopoietic	3–4	19	17.06	1.11	0.71	1.75
Malignant_hematopoietic	>=5	23	18.20	1.26	0.84	1.90
Multiple_myelomas	<1	5	1.47	3.40	1.41	8.16
Multiple_myelomas	1–2	6	4.06	1.48	0.66	3.29
Multiple_myelomas	3–4	4	2.52	1.59	0.60	4.24
Multiple_myelomas	>=5	3	2.75	1.09	0.35	3.38
Non-Hodgkin_lymphoma	<1	6	3.22	1.86	0.84	4.14
Non-Hodgkin_lymphoma	1–2	12	8.70	1.38	0.78	2.43
Non-Hodgkin_lymphoma	3–4	4	5.35	0.75	0.28	1.99
Non-Hodgkin_lymphoma	>=5	6	5.74	1.05	0.47	2.33
Oesophagus	<1	3	1.26	2.39	0.77	7.41
Oesophagus	1–2	7	3.47	2.02	0.96	4.24
Oesophagus	3–4	3	2.15	1.39	0.45	4.32
Oesophagus	>=5	6	2.32	2.59	1.16	5.77
Other_illdefined	<1	8	1.68	4.75	2.37	9.49
Other_illdefined	1–2	7	4.23	1.66	0.79	3.47
Other_illdefined	3–4	3	2.41	1.25	0.40	3.86
Other_illdefined	>=5	2	2.28	0.88	0.22	3.51
Pancreas	<1	4	2.27	1.77	0.66	4.70
Pancreas	1–2	11	6.30	1.75	0.97	3.15
Pancreas	3–4	6	3.99	1.50	0.68	3.35

Cancer. site	Follow–up time, years	N observed	N expected	SIR	lCI95	uCI95
Pancreas	>=5	3	4.41	0.68	0.22	2.11
Prostate	<1	38	36.83	1.03	0.75	1.42
Prostate	1–2	54	98.61	0.55	0.42	0.71
Prostate	3–4	43	59.94	0.72	0.53	0.97
Prostate	>=5	41	62.26	0.66	0.48	0.89
Skin_melanoma	<1	12	5.27	2.28	1.29	4.01
Skin_melanoma	1–2	25	14.82	1.69	1.14	2.50
Skin_melanoma	3–4	15	9.36	1.60	0.97	2.66
Skin_melanoma	>=5	17	10.41	1.63	1.02	2.63
Skin_nonmelanoma	<1	51	10.07	5.07	3.85	6.67
Skin_nonmelanoma	1–2	63	28.89	2.18	1.70	2.79
Skin_nonmelanoma	3–4	46	18.07	2.55	1.91	3.40
Skin_nonmelanoma	>=5	44	20.16	2.18	1.62	2.93
Stomach	<1	6	1.93	3.12	1.40	6.94
Stomach	1–2	8	5.09	1.57	0.79	3.14
Stomach	3–4	10	3.07	3.26	1.75	6.05
Stomach	>=5	4	3.21	1.24	0.47	3.32

CNS, central nervous system

TABLE E 7. Site–specific standardized incidence ratios (SIR) of cancer by length of follow up for women. SIRs are presented with lower (lCI95) and upper (uCI95) limits of 95% confidence intervals. Cancers with >15 cases are shown.

Cancer.site	Follow–up time, years	N observed	N expected	SIR	lCI95	uCI95
Urinary bladder	<1	2	1.17	1.70	0.43	6.82
Urinary bladder	1–2	6	4.47	1.34	0.60	2.99
Urinary bladder	3–4	4	3.16	1.26	0.47	3.37
Urinary bladder	>=5	4	1.45	2.75	1.03	7.34
Breast	<1	14	9.38	1.49	0.88	2.52
Breast	1–2	32	33.66	0.95	0.67	1.34
Breast	3–4	26	23.33	1.11	0.76	1.64
Breast	>=5	19	27.30	0.70	0.44	1.09
Cervix_uteri	<1	6	0.46	13.17	5.92	29.31
Cervix_uteri	1–2	11	1.66	6.62	3.67	11.95
Cervix_uteri	3–4	6	1.19	5.06	2.27	11.26
Cervix_uteri	>=5	9	1.48	6.09	3.17	11.71
Colorectal	<1	12	4.63	2.59	1.47	4.56
Colorectal	1–2	24	17.06	1.41	0.94	2.10
Colorectal	3–4	17	11.90	1.43	0.89	2.30
Colorectal	>=5	14	14.28	0.98	0.58	1.65
Corpus_uteri	<1	8	2.22	3.60	1.80	7.21
Corpus_uteri	1–2	5	7.82	0.64	0.27	1.54
Corpus_uteri	3–4	6	5.34	1.12	0.50	2.50
Corpus_uteri	>=5	4	6.26	0.64	0.24	1.70
Lip_oral_cavity_pharynx	<1	5	0.60	8.37	3.48	20.11
Lip_oral_cavity_pharynx	1–2	6	2.26	2.66	1.19	5.92
Lip_oral_cavity_pharynx	3–4	4	1.61	2.49	0.93	6.63
Lip_oral_cavity_pharynx	>=5	2	3.85	0.52	0.13	2.08
Lung	<1	14	3.35	4.18	2.48	7.06
Lung	1–2	26	12.35	2.11	1.43	3.09
Lung	3–4	9	8.58	1.05	0.55	2.02
Lung	>=5	14	10.32	1.36	0.80	2.29
Malignant_hematopoietic	<1	13	3.09	4.20	2.44	7.24
Malignant_hematopoietic	1–2	21	11.43	1.84	1.20	2.82
Malignant_hematopoietic	3–4	12	8.01	1.50	0.85	2.64
Malignant_hematopoietic	>=5	13	9.66	1.35	0.78	2.32
Multiple_myelomas	<1	5	0.68	7.40	3.08	17.77
Multiple_myelomas	1–2	6	2.46	2.44	1.10	5.43
Multiple_myelomas	3–4	4	1.64	2.44	0.92	6.50
Multiple_myelomas	>=5	3	2.54	1.18	0.38	3.66
Multiple_myelomas 0	>=5	3	1.90	1.58	0.51	4.89
Non-Hodgkin_lymphoma	<1	6	1.55	3.87	1.74	8.62
Non-Hodgkin_lymphoma	1–2	12	5.52	2.17	1.23	3.83
Non-Hodgkin_lymphoma	3–4	4	3.73	1.07	0.40	2.86
Non-Hodgkin_lymphoma	>=5	6	4.38	1.37	0.62	3.05
Other_illdefined	<1	7	1.16	6.02	2.87	12.62
Other_illdefined	1–2	9	3.81	2.36	1.23	4.54
Other_illdefined	3–4	8	2.50	3.20	1.60	6.40
Other_illdefined	>=5	2	2.70	0.74	0.19	2.96
Pancreas	<1	2	1.01	1.99	0.50	7.94
Pancreas	1–2	10	3.86	2.59	1.39	4.82
Pancreas	3–4	6	2.73	2.20	0.99	4.89
Pancreas	>=5	1	3.37	0.30	0.04	2.11

Cancer. site	Follow–up time, years	N observed	N expected	SIR	lCI95	uCI95
Skin_melanoma	<1	7	1.94	3.61	1.72	7.58
Skin_melanoma	1–2	11	7.49	1.47	0.81	2.65
Skin_melanoma	3–4	8	5.48	1.46	0.73	2.92
Skin_melanoma	>=5	12	6.89	1.74	0.99	3.07
Skin_nonmelanoma	<1	27	2.94	9.20	6.31	13.41
Skin_nonmelanoma	1–2	44	11.78	3.74	2.78	5.02
Skin_nonmelanoma	3–4	26	8.75	2.97	2.02	4.37
Skin_nonmelanoma	>=5	22	10.73	2.05	1.35	3.11

CNS, central nervous system
